# Health and human rights: a statistical measurement framework using household survey data in Uganda

**DOI:** 10.1186/s12914-015-0049-8

**Published:** 2015-05-03

**Authors:** Ronald Wesonga, Abraham Owino, Agnes Ssekiboobo, Leonard Atuhaire, Peter Jehopio

**Affiliations:** School of Statistics and Planning, Makerere University, Kampala, Uganda

**Keywords:** Human rights, Health indicators, Statistical models, Household survey

## Abstract

**Background:**

Health is intertwined with human rights as is clearly reflected in the right to life. Promotion of health practices in the context of human rights can be accomplished if there is a better understanding of the level of human rights observance. In this paper, we evaluate and present an appraisal for a possibility of applying household survey to study the determinants of health and human rights and also derive the probability that human rights are observed; an important ingredient into the national planning framework.

**Methods:**

Data from the Uganda National Governance Baseline Survey were used. A conceptual framework for predictors of a hybrid dependent variable was developed and both bivariate and multivariate statistical techniques employed. Multivariate post estimation computations were derived after evaluations of the significance of coefficients of health and human rights predictors.

**Results:**

Findings, show that household characteristics of respondents considered in this study were statistically significant (p < 0.05) to provide a reliable assessment of human rights observance. For example, a unit increase of respondents’ schooling levels results in an increase of about 34% level of positively assessing human rights observance. Additionally, the study establishes, through the three models presented, that household assessment of health and human rights observance was 20% which also represents how much of the entire continuum of human rights is demanded.

**Conclusion:**

Findings propose important evidence for monitoring and evaluation of health in the context human rights using household survey data. They provide a benchmark for health and human rights assessments with a focus on international and national development plans to achieve socio-economic transformation and health in society.

## Background

Health is intertwined with human rights as is clearly reflected in the right to life which is a basis for enjoyment of all other human rights. Promotion of health practices in the context of human rights can be accomplished if there is a better understanding of the level of human rights observance. Human rights indicators are therefore critical to the health of society and the national development agenda and can be classified into three categories namely; i) structural indicators that reflect the ratification or adoption of legal instruments and existence of basic institutional mechanisms deemed necessary for facilitating realisation of the concerned human right; ii) process indicators that relate the state policy instruments with milestones which accumulate into outcomes that can be more directly related to realisation of a right, hence capture accountability as well as the notion of progressive realisation; and iii) outcome indicators that capture attainments, individual and collective, and reflect the status of realisation of the human rights in a given context [1-4]. Statistics on the other hand renders a scientific approach to the development, assessment and monitoring of human rights indicators [5,6]. Hitherto, accuracy in developing statistically reliable human rights indicators has not been given the attention it deserves, probably not because of the process complexity, but maybe because of the fact that it is an assumed psychometric area of statistical application.

However, initiatives have been undertaken to scientifically conceptualise human rights so as to develop statistically reliable human rights indicators. For instance, there are four main categories and three dimensions of human rights. The four categories are; economic, political, social and cultural (EPSC) rights while the three dimensions are; respect, protect and fulfil [1,7,8]. These categorisations form the fulcrum of statistical measurement of human rights [7,9-11]. Furthermore, the initiatives so far taken towards development of human rights indicators could be categorised as; event-based data on human rights violations, socio-economic and administrative statistics, household perceptions and opinion surveys, and expert judgements [4,12]. In response to these requirements, Uganda conducted the first ever governance baseline survey that among other themes collected data on human rights to inform the national development agenda [13-15]. In this paper, we assess the determinants of human rights and develop models for measuring human rights observance at the national-level that would be a source for monitoring health and socio-economic development goals. Subsequently, the following propping questions might be answered: What are the key determinants of human rights? Are there variations in the parameters that explain human rights? How much of the human rights continuum do the identified parameters explain? Are there variations in the different models that explain human rights observance?

Three core aspects namely; definition, measurement and prediction have greatly affected appreciation and localisation of human rights in the political, cultural, economic and social spheres of humanity [16,17]. The complexity to human rights stems from the level of difficulty to define human rights whereas the failure to make reliable prediction could be due to unavailability of a reliable approach to measure and monitor the human rights [18,19]. A number of scholars [20,4,21] have attempted to provide some definitions, but to-date there is no single definition of human rights that is acceptable as a standard by all those fighting for human rights. Surprisingly, in this modern era, there are still some sections of humanity and other cultures that do not believe, for example that women are human beings and as such should not prescribe to human rights [22,8,23]. In his book, [24] contends that although a human right is a strong idea, it is often used loosely and can have different meanings in different contexts and those who use the idea so readily seldom stop to ponder its various meanings and contradictions. Nonetheless, human rights are described as universal legal guarantees that protect the fundamental freedoms and human dignity of every individual [16,10,2,21]. They affirm that every human being is entitled to equal treatment and opportunities regardless of belonging to any section of society.

It is often said that to manage, you must be able to measure, conversely, what you cannot measure, you cannot manage. For human rights to be understood, defended, localised in promotion of health outcomes for the deprived societies, measurable indicators must be developed to reflect the local context of a region or country [11]. It is imperative that such indicators be developed to cover the five categories of human rights; civil, economic, political, social and cultural rights against the three dimension of respect, protect and fulfil.

**Figure 1:**
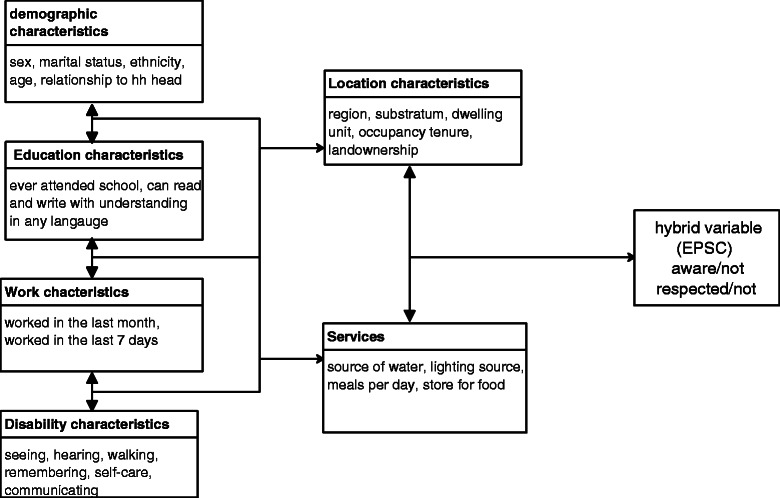
Conceptual framework for determinants of health and human rights.

Theoretically, a household is at the core of health and human rights assessments because it is within a household that humans live. Therefore, to understand health and human rights and be able to assess and predict them, the theoretical and conceptual designs have to focus at a household as a data source [25]. In this context, a national governance baseline survey carried out with household as the unit of analysis, yielded the desired data for assessing human rights. The Uganda National Governance Baseline Survey was carried out with a focus on five key themes of governance, among which was the theme on human rights, details on this will be presented in the section on data sources and description and more details can be found in the report [26]. Theoretically, it is well conceptualised that the status of health and well-being of a person is premised on the degree to which he enjoys the human rights [13,27,28]. The more the human rights are denied or the tendency therein, the less likely that such a person is happy and healthy. In this regard, a dependent variable that aggregates the three dimensions of respect, protect and fulfil, was thus, developed to take cognizance of the three dimensions of human rights. The composite indicator variable is adequately described in the preceding sections. To assess levels of human rights adequately at household level depends on the level of awareness about the economic, political, social, cultural and civil human rights. Therefore, one can at statistical confidence level tell whether, human rights are respected or not. However, there are variations in degrees of awareness and even respect for human rights by respondent’s demographic, educational, employment and disability characteristics which are also moderated by location and availability of services in their localities as shown in Figure 1.

Violation of one’s right to housing leads to violation of various other rights because these rights are inter-related [29-31]. According to UNHCHR [31], “Inadequate housing can have repercussions on the right to health; for instance, if houses and settlements have limited or no safe drinking water and sanitation, their residents may fall seriously ill.” Inadequate housing may also imply lack of or poor food storage facilities thus affecting food availability, food access and food quality [32]. UNHCHR [31] also notes that the right to adequate housing can be affected by the extent to which other human rights are guaranteed, for instance, access to housing is most at risk for those denied the right to education, work or social security. Employment status, education and dwelling decency are among the variables considered in this study. Quality of housing is strongly associated with wellbeing as shown by the fact that inadequate housing increases the risk of severe ill health and disability and even poor mental health. It is also associated with lower educational attainment, unemployment and poverty [33,34]. The UN Committee [35] stated that housing must provide adequate shelter, which means adequate privacy, space, security, lighting and ventilation, basic infrastructure and location with regard to work and basic facilities, all at a reasonable cost. The variables that focus on the right to housing (housing condition, status and location) in this study include rural/urban and regional locations, decency of the dwelling unit, ownership status for both house and land and source of lighting. The Convention on the Rights of the Child [36] also stipulates that everyone should have a right to sustainable access to natural and common resources, safe drinking water, energy for cooking, heating and lighting, sanitation and washing facilities, means of food storage, refuse disposal, site drainage and emergency services. In this study the variables considered from this list were source of water, meals per day and granary status for food storage.

## Methods

### Data sources and description

The data used in this paper were derived from the Uganda National Governance Baseline Survey (UNGBS) conducted by the Uganda Bureau of Statistics in collaboration with Makerere University, School of Statistics and Planning, with support from the United Nations Development Programme. A national sample of 4776 households was scientifically drawn and data collected on various themes of governance among which was the theme on human rights. Table 1 shows eighteen variables that were carefully chosen for this study, whereof the last variable, human rights respect, *hr_hybrid* is the dependent variable of the models developed. This variable was developed to capture both aspects of knowledge of a human right and being able to assess the observance of human rights [37]. In most developing countries and largely so, many citizens may not be aware of some of their rights! Therefore, a condition was developed to the effect that if one is not knowledgeable enough about some right, then that person does not have a sufficient ground to assess the level of observance of such a right. Thus, the development of a hybrid dependent variable that reliably measures both knowledge and assessment of human rights [38].

**Table 1:** **Description of variables for the framework**

Variable	Variable description
Urbanrural	urban or rural residence
Region	Region
Age	age of respondent
Hhhead	head of household
Mstatus	marital status
Readwrite	can read and write
Attendschool	ever attended school
Employstatus	employment status
Dwelling	dwelling unit decency status
Ownhouse	house ownership status
Watersource	source of water
Lighting	source of lighting
Ownland	land ownership status
Granary	granary presence
Sex	sex of respondent
Disability	disability status
Meals	meals per day
hr_hybrid	Human rights respect

### Framework for health and human rights level of observance

A conceptual framework was developed to show the relationship that exists between the independent variables and the hybrid dependent variable for the study as shown in Figure 1. The hybrid dependent variable was derived such that it covered knowledge and assessment aspects of economic, political, social and cultural human rights with health treated as a crosscutting indicator. For example, knowledge on the right to seek for justice under the law, the right of victims, suspects, accused persons, prisoners, and the right to vote, access information, among others were considered. The independent variables used in the modelling process are primarily for the typical citizens’ characteristics, intervened by location and services’ characteristics [39,27,5,40]. Table 1 shows a description of variables used to assess health and human rights at household level. Specific independent variables were categorised under demographic, educational, work and disability characteristics. The other categories, location and availability of services were used as intervening variables. The purpose was to explore the effect of belonging to a certain category on assessment of the level of health and human rights observance.

The fact that the stochastic structure of the data are expressed in terms of Bernoulli and Binomial distributions; whereby the hybrid dependent variable (*hr_hybrid)* bore two categories (aware/respected vis-a-vis not aware/respected), implied the application of binary logistic regression modelling approach [41,42]. Accordingly, the conceptual framework implied development and comparison of three logit models. In the logistic regression model, the random variable Y_*i*_ is assumed to have a binomial distribution;

1 $$ {Y}_i\sim Binomial\left({n}_i,{\pi}_i\right) $$

which then defines the stochastic structure of the model with a binomial denominator *n*_*i*_ and probability *π*_*i*_ We suppose that the logit of the underlying probability *π*_*i*_ is a linear function of the predictors for human rights as shown in Figure 1, then;

2 $$ logit\left({\pi}_i\right)={X}_i^{\hbox{'}}\beta $$

Where *X*_*i*_ a vector of covariates for human rights and β is a vector of regression coefficients for the covariates. Thus, Equations (1) and (2) define generalised linear models for determinants of human rights with binomial response and logit link [43,44]. The findings presented in Table 2 for the three models are each derivatives of the logit model described as in Equation 2.

**Table 2:** **Factors associated with health and human rights (knowledge and assessment)**

Health and human rights observance (knowledge and assessment)					
	HR not respect		HR respected		Test statistic
	Percent	95 Percent CI	Percent	95 Percent CI	χ ^ 2 ^ /F/p-value
**Region**					
Kampala	74.8	[70.9-78.3]	25.2	[21.7-29.1]	
Central	87.6	[85.1-89.7]	12.4	[10.3-14.9]	
Eastern	80.8	[77.5-83.7]	19.2	[16.3-22.5]	
Northern	79.3	[76.2-82.0]	20.7	[18.0-23.8]	χ^2^ (4) = 40.7943; Design-based F(3.48, 16594.36) = 7.6073 **P = 0.000**
Western	83.5	[80.5-86.1]	16.5	[13.9-19.5]	
Total	82.6	[81.2-83.9]	17.4	[16.1-18.8]	
**Urban or Rural residence**					
Urban	78.6	[75.0-81.8]	21.4	[18.2-25.0]	χ^2^ (1) = 14.7354; Design-based F(1.00, 4770.00) = 8.3113 **P = 0.004**
Rural	83.7	[82.2-85.1]	16.3	[14.9-17.8]	
Total	82.6	[81.2-83.9]	17.4	[16.1-18.8]	
**Sex of respondent**					
Male	77.6	[75.3-79.7]	22.4	[20.3-24.7]	χ^2^ (1) = 67.4419; Design-based F(1.00, 4770.00) = 41.6579 **P = 0.000**
Female	86.7	[84.9-88.3]	13.3	[11.7-15.1]	
Total	82.6	[81.2-83.9]	17.4	[16.1-18.8]	
**Head of household**					
not head	85.4	[83.4-87.3]	14.6	[12.7-16.6]	χ^2^ (1) = 19.3278; Design-based F(1.00, 4770.00) = 12.0582 **P = 0.001**
Head	80.5	[78.6-82.3]	19.5	[17.7-21.4]	
Total	82.6	[81.2-83.9]	17.4	[16.1-18.8]	
**Marital status**					
Other	84.9	[81.3-87.9]	15.1	[12.1-18.7]	
Single	85.8	[81.8-89.0]	14.2	[11.0-18.2]	χ^2^ (2) = 8.1345; Design-based F(1.98, 9446.38) = 2.7399 **P = 0.065**
Married	81.7	[80.0-83.3]	18.3	[16.7-20.0]	
Total	82.6	[81.2-83.9]	17.4	[16.1-18.8]	
**Ever attended school**					
Never attended school	89.6	[87.1-91.7]	10.4	[8.3-12.9]	χ^2^ (1) = 41.4547; Design-based F(1.00, 4770.00) = 28.0364 **P = 0.000**
Attended school	80.8	[79.2-82.4]	19.2	[17.6-20.8]	
Total	82.6	[81.2-83.9]	17.4	[16.1-18.8]	
**Can read and write**					
Unable to read and write	87.9	[85.8-89.7]	12.1	[10.3-14.2]	χ^2^ (1) = 52.2933; Design-based F(1.00, 4770.00) = 32.8182 **P = 0.000**
Able to read at least	79.6	[77.8-81.4]	20.4	[18.6-22.2]	
Total	82.6	[81.2-83.9]	17.4	[16.1-18.8]	
**Employment status**					
Not employed	84.6	[82.0-87.0]	15.4	[13.0-18.0]	
Employed	81.8	[80.1-83.4]	18.2	[16.6-19.9]	χ^2^ (1) = 5.3350; Design-based F(1.00, 4770.00) = 3.2375 **P = 0.072**
Total	82.6	[81.2-83.9]	17.4	[16.1-18.8]	
**Disability status**					
Disabled	83.6	[81.5-85.4]	16.4	[14.6-18.5]	
Not disabled	81.7	[79.7-83.5]	18.3	[16.5-20.3]	χ^2^ (1) = 2.9718; Design-based F(1.00, 4770.00) = 1.8346 ***P = 0.176***
Total	82.6	[81.2-83.9]	17.4	[16.1-18.8]	
**Dwelling unit decency status**					
Not decent	84.0	[82.0-85.9]	16	[14.1-18.0]	
Decent dwelling	81.4	[79.4-83.2]	18.6	[16.8-20.6]	chi2(1) = 5.7662; Design-based F(1.00, 4770.00) = 3.6264 ***P = 0.057***
Total	82.6	[81.2-83.9]	17.4	[16.1-18.8]	
**House ownership status**					
Not own/free	81.1	[77.5-84.3]	18.9	[15.7-22.5]	
Own/free	82.9	[81.4-84.4]	17.1	[15.6-18.6]	χ^2^ (1) = 1.6285; Design-based F(1.00, 4770.00) = 0.9812 ***P = 0.322***
Total	82.6	[81.2-83.9]	17.4	[16.1-18.8]	
**Source of water**					
Public	82.7	[81.1-84.2]	17.3	[15.8-18.9]	
Private	77.4	[70.4-83.2]	22.6	[16.8-29.6]	χ^2^ (2) = 7.6536; Design-based F(1.96, 9343.03) = 2.1905 ***P = 0.113***
Protected	84.3	[80.8-87.2]	15.7	[12.8-19.2]	
Total	82.6	[81.2-83.9]	17.4	[16.1-18.8]	
**Source of lighting**					
Paraffin	83.1	[81.4-84.7]	16.9	[15.3-18.6]	
Public	78.3	[74.2-81.9]	21.7	[18.1-25.8]	χ^2^ (2) = 9.2866; Design-based F(1.98, 9460.88) = 2.9893 ***P = 0.051***
Private	83.6	[80.4-86.5]	16.4	[13.5-19.6]	
Total	82.6	[81.2-83.9]	17.4	[16.1-18.8]	
**Land ownership status**					
Do not own land	82.7	[79.7-85.4]	17.3	[14.6-20.3]	
Own land	82.6	[81.0-84.1]	17.4	[15.9-19.0]	χ^2^ (1) = 0.0129; Design-based F(1.00, 4770.00) = 0.0083 ***P = 0.928***
Total	82.6	[81.2-83.9]	17.4	[16.1-18.8]	
**Meals per day**					
Less than three meals	83.9	[82.2-85.4]	16.1	[14.6-17.8]	
Three or more meals	79.4	[76.7-81.9]	20.6	[18.1-23.3]	χ^2^ (1) = 13.6498; Design-based F(1.00, 4770.00) = 8.7547 ***P = 0.003***
Total	82.6	[81.2-83.9]	17.4	[16.1-18.8]	
**Granary availability**					
No granary	83.2	[81.6-84.8]	16.8	[15.2-18.4]	χ^2^ (1) = 3.1387; Design-based F(1.00, 4770.00) = 1.9306 ***P = 0.165***
Has granary	81.1	[78.4-83.6]	18.9	[16.4-21.6]	
Total	82.6	[81.2-83.9]	17.4	[16.1-18.8]	

### Ethical consideration

This study used secondary data collected by the Uganda Bureau of Statistics, which is entitled by Ugandan law to collect and disseminate official statistics. The data were anonymised so as to conceal the identity of the household respondents, as is the required.

## Results

### Relationship between health and human rights observance and its covariates

Table 2 presents the bivariate analysis to establish the relationships between human rights observance and its explanatory variables. Using the chi-squared and the design-based F-tests, the relationship between eight of the seventeen candidate variables and human rights observance were statistically significant (*p < 0.05*), four variables were marginally statistically significant (*p ≈ 0.05*) while five variables were statistically not significant (*p > 0.05*).

### Models for determinants of health and human rights observance

Table 3 presents three multivariate statistical models that explain human rights observance at the national level. The difference between the three models is the number of predictors for the hybrid human rights observance as provided in the conceptual framework in Figure 1. The models were developed such that model one contained all the preconceived variables necessary for assessing level of observance of human rights. Model two was constructed such that it excludes the four location variables (*urbanrural, dwelling, ownhouse and ownland*) whereas model three excluded the four services-based variables (*watersource, lighting, granary and meals*) besides the location variables. This was designed so as to study the model dynamics for changes in assessment of human rights observance when influential characteristics of location and service provision are controlled.

**Table 3:** **Determinants of health and human rights (knowledge and assessment) at national level**

Dependent: Human rights status	HR Model one OR (S.E)	HR Model two OR (S.E)	HR Model three OR (S.E)
**Sex of respondent**			
Male	1.000	1.000	1.000
Female	0.610** (0.001)	0.621** (0.001)	0.617** (0.001)
**Head of household**			
Not head	1.000	1.000	1.000
Head	1.077** (0.003)	1.081** (0.003)	1.066** (0.002)
**Marital status**			
Single	1.000	1.000	1.000
Married	1.075** (0.001)	1.076** (0.001)	1.076** (0.001)
**Ever attended school**			
Never attended school	1.000	1.000	1.000
Attended school	1.343** (0.004)	1.342** (0.004)	1.356** (0.004)
**Can read and write**			
Unable to read and write	1.000	1.000	1.000
Able to read at least	1.362** (0.003)	1.435** (0.004)	1.456** (0.004)
**Employment status**			
Not employed	1.000	1.000	1.000
Employed	1.078** (0.002)	1.087** (0.002)	1.074** (0.002)
**Disability status**			
Disabled	1.000	1.000	1.000
Not disabled	0.952** (0.002)	0.973** (0.002)	0.982** (0.002)
**Age**	1.000** (0.000)	1.001** (0.000)	1.001** (0.000)
**Meals per day**			
less than three meals	1.000	1.000	
three or more meals	1.217** (0.002)	1.268** (0.002)	
**Granary availability**			
No granary	1.000	1.000	
Nas granary	1.143** (0.002)	1.151** (0.002)	
**Source of water**			
Public	1.000	1.000	
Private	0.925** (0.001)	0.946** (0.001)	
**Source of lighting**			
Public	1.000	1.000	
Private	0.995** (0.001)	0.997** (0.001)	
**Land ownership status**			
Do not own land	1.000		
Own land	1.021** (0.003)		
**House ownership status**			
Not own/free	1.000		
Own/free	0.951** (0.003)		
**Dwelling unit decency status**			
Not decent	1.000		
Decent dwelling	1.199** (0.002)		
**Urban or Rural residence**			
Urban	1.000		
Rural	0.874** (0.001)		
**Model diagnostic checks**			
Number of households	4771	4771	4771
Log-likelihood	−4.19E + 06	−4.20E + 06	−4.22E + 06
Degrees of freedom	16	12	8
Akaike information criterion	8.39E + 06	8.41E + 06	8.43E + 06
Bayesian information criterion	8.39E + 06	8.41E + 06	8.43E + 06

Note: Odds Ratio (OR); standard error (S.E) in parentheses; (+ = p < 0.10, * = p < 0.05, ** = p < 0.01); for completeness, the 1.000 represent base categories.

It was observed that for all variables that significantly predicated level of observance of human rights in the three models bore a consistent behaviour throughout the modelling cycle. The odds for reporting that human rights are respected were greater for the respondents who were heads of households, married, ever attended school, could read and write, employed, had three or more meals per day, had a granary, owned land and dwelled in a decent unit. Notably, the odds for reporting \that human rights are respected were less for respondents; who were female, had no disability, had private sources of water and lighting, owned a house and resided in a rural area. Accordingly, the entire model diagnostic tests (Log-likelihood ratio test, Akaike information criterion and Bayesian information criterion) shown recommend model one as the most reliable and coherent human rights observance predictor model.

**Figure 2:**
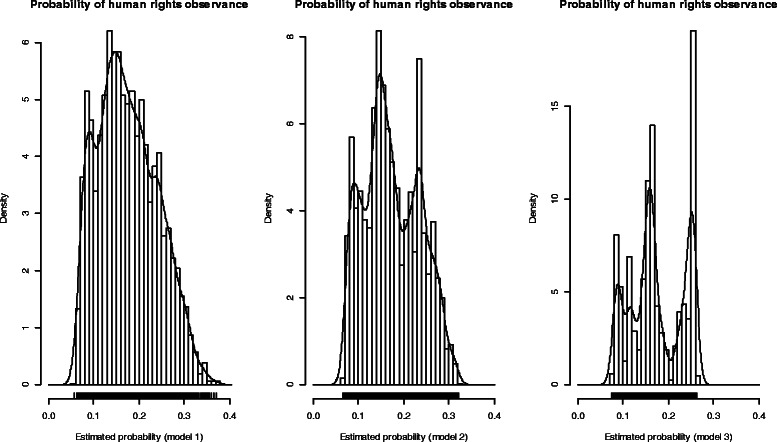
Multivariate model-based probability of health and human rights observance.

Figure 2 shows the probabilities for the levels of prediction of human rights by the different covariates based on estimates from models one, two and three presented in Table 2. Furthermore, using the graphical evidence, it is confirmed that model one portrayed the best estimate of human rights observance followed by model two and model three respectively. Model one is one which utilised all variables in its estimation of human rights observance. The level of reliability of estimates for human rights observance could also be determined from the plot’s proximity to the normal distribution. Overall, the proportion of the human rights observance that was estimated using any of the three models is about 20 percent that may stretch up to a maximum of 40 percent for model one.

## Discussions

The two strands derived from the findings support the conceptual framework presented in this study. Firstly, the multivariate statistical model analyses show that the model with a complete set of covariates as suggested provides the best fit to the baseline survey according to the diagnostic tests (Log-likelihood ratio test, Akaike information criterion and Bayesian information criterion) presented in Table 2. Having passed the goodness of fit test using Hosmer-Lemeshow test, further tests were performed to establish the best model among the three proposed. The model with the best fit was identified as one with the smallest value for anyone of the three diagnostic tests applied. All the three model candidates show the exponentiated coefficients, commonly referred to as the odds ratios. These odds ratios, though, do not contradict for any one model, they present intrinsic differences. For example, female household respondents are less likely to report that human rights are observed than the male counterparts by about 39 percent. The other determinants with negative odds include; female respondents, households with private water source, households with private lighting source, respondents who own land and those who stay in rural areas. Respondents who are heads of households are more likely to report that human rights are observed than those respondents who are not heads of households by about 8 percent. The other determinants with positive odds include; head of household, those who attended school, those who can read and write, those who are employed, those who can afford three or meals per day, those with a granary, those who own land and those with decent dwelling. The best model fit tends to present a more optimal position for evaluating the level of human rights observance, hence the health status of households and the communities as a whole. Secondly, the bivariate plot, Figure 2 shows that models 2 and 3 greatly distort the known normal distribution curve *N*(*μ =* 0*; σ =* 1) that leaves model 1 as the best fit, thus may not adequately be used to determine the level of health and human rights adherence [17].

From the theoretical perspective, health and human rights present to the development and health planners a very intricate scenario that is sometimes difficult to quantify [11,45,46]. However, principally, human rights observance presents two components; the indelible rights which are more permanent and hard to continuously deny, for example the right to life and the provisional rights which are transitory or short-lived, for example the right to access electoral or public information [47,1,48,49]. Findings from this study (Figure 2; model one) show that the indelible human rights represent at least 60 percent of the overall human rights; these rights were not measured, but were implied from the best human rights observance *model one* that estimated the provisional human rights to be about 40 percent. This is a very significant contribution not only to the literature of human rights, but also to the wealth of knowledge of statistical, health and development studies involving human rights observance levels.

Furthermore, it can still be shown that judgement or assessment of health and human rights, where only respondents are involved, will on average be 20 percent of the entire human rights observance assessment. The argument for this conclusion is that while assessing human rights, there are two sides; the demand side and the supply side. Therefore, interaction with a typical citizen as an assessor will lead to an average estimate of 20 percent as shown in Figure 1 of this study. This is one of the main limitations of assessing human rights using household survey data whose acknowledgement could improve reliability of human rights assessments.

## Conclusions

In summary, the study explored the possibility of developing a system of assessing levels of human rights in the context of health based using household survey methodology. The key determinants of health and human rights were established using survey data. Accordingly all variables proposed in the study were significant (*p < 0.01*), as they generated the best model (*model 1*) to estimate national-level human rights observance. Seemingly, at bivariate level, using the χ^2^ − *test* and design-based *F-test* statistics, there were slight variations in the associations of the hybrid variable and parameters that explained it. Findings show that to assess health and human rights observance, the household survey methodology approach yields an average of 20 percent of the entire human rights continuum as revealed by the three different human rights observance models presented in this study. Ironically, suggesting that 20 percent is demanded from governments, the other 20 percent are on the supply side as protected through constitutions and the biggest percentage (60%) are the indelible human rights, God-given and protected. Higher probabilities of observance imply better levels of health and livelihoods experienced. Lastly, findings of this study are instrumental in developing and harmonising health, human rights and national development.
